# Atorvastatin does not display an antimicrobial activity on its own nor potentiates the activity of other antibiotics against *Acinetobacter baumannii* ATCC17978 or *A. baumannii* AB030

**DOI:** 10.1099/acmi.0.000288

**Published:** 2021-11-30

**Authors:** Vanessa Kornelsen, Mark Unger, Ayush Kumar

**Affiliations:** ^1^​ University of Manitoba, Winnipeg, Manitoba, Canada

**Keywords:** drug repurposing, multidrug resistance, drug development, Gram-negative

## Abstract

With the current arsenal of antibiotics increasingly becoming ineffective against bacteria, there is an increasing interest in the possibility of using previously approved non-antibiotic drugs as antimicrobials. Statins have recently been investigated for their antimicrobial activity and their ability to potentially synergize with current treatment options. Atorvastatin had been shown previously to be the most promising candidate for effectivity against *Acinetobacter baumannii* ATCC17978. In this study, we tested atorvastatin for its activity against an extensively drug-resistant (XDR) strain *A. baumannii* AB030. However, our data show that atorvastatin has no effect *A. baumannii* AB030. Intriguingly, atorvastatin was also ineffective against our laboratory’s *A. baumannii* ATCC17978. This lack of atorvastatin activity against *A. baumannii* ATCC17978 cannot be attributed to RND efflux pumps as a strain deficient in the three most clinically relevant RND efflux systems in *A. baumannii* showed no change in susceptibility compared to its parent strain ATCC17978. Further, atorvastatin failed to potentiate the activity of tobramycin and ciprofloxacin. While it is not clear to us why atorvastatin is not active against *A. baumannii* ATCC17978 used in our study, our study shows that evaluation of compounds for their antibacterial activity should involve multiple strains to account for strain-to-strain variation.

## Introduction

*Acinetobacter baumannii* is an opportunistic pathogen that mainly causes hospital-acquired infections such as skin and soft tissues infections, ventilator-associated pneumonia, catheter-associated urinary tract infections, and bacteremia [1]. The infections caused by this bacterium can be difficult to treat due to its high genomic plasticity and its intrinsic and adaptive resistance to antimicrobials [2]. This has led to increased instances in multidrug-resistant clinical isolates and the World Health Organization has placed *A. baumannii* at the top of its list of bacterial species requiring increased research, discovery and development of new treatment options [3]. Current treatment options are often limited and carbapenems are the drug of choice [4]. If carbapenem resistance is seen then few options remain, with tigecycline and polymyxins being the antibiotics of last resort [4]. Combination therapy is preferred to ensure clearance of the infection, especially if the infectious agent has not been clearly identified, and often carbapenems or polymyxins will be combined with antipseudomonal antibiotics such as aminoglycosides and fluoroquinolones to improve outcomes [5]. With this dwindling supply of treatment options, researchers are looking for new treatments but also investigating old treatments for clearing *A. baumannii* infections. One such approach is to repurpose previously approved non-antibiotic drugs for use as antimicrobial agents.

Statins are traditionally used to control cholesterol levels, specifically they lower lipid levels by inhibiting 3-hydroxy-3-methylglutaryl coenzyme A (HMG-CoA) [6]. Interestingly it was found that statins had a larger effect on preventing coronary heart disease than simply changing lipid levels would suggest and as such further investigations revealed that these compounds are able to have pleiotropic effects on the human body [7]. Statins appear to play a role in decreasing inflammation and oxidative stress, increasing the stability of atherosclerotic plaques and improving endothelial function, to name a few [7]. Additionally, studies had shown that patients taking statins at the time of developing pneumonia were less likely to have serious complications, develop sepsis, or die from sepsis [8]. This was suggesting that perhaps these compounds may also exhibit antimicrobial activity and thus lead to these improved pneumonic outcomes. However there are also suggestions that the improved patient outcome may be due to the pleiotropic effects of statins on the host itself, such as immunomodulation, especially reduction in inflammation [7]. There is also evidence that statins can disrupt isoprenoid metabolism, particularly in Gram-positive bacteria, and therefore lead to reduced stability within membranes [9, 10]. Disturbance of the outer membrane of Gram-negative bacteria is also seen with aminoglycosides such as tobramycin and the potential for a similar mode of action regarding the bacterial membrane could be investigated.

A previous study determined the susceptibility of three statins (simvastatin, rosuvastatin, and atorvastatin) for a variety of clinically relevant bacterial species, including *A. baumannii* [11]. The most promising statin in regard to antimicrobial activity appeared to be atorvastatin as it was determined to have the lowest MIC values of the three compounds against the largest number of strains. The purpose of our study was to evaluate the activity of atorvastatin against pandrug resistant isolate *A. baumannii* AB030.

## Methods

### Bacterial strains and growth conditions

All relevant strains and plasmids are listed in Table 1 and primers used are listed in Table 2. Overnight cultures for all strains were grown in LB broth, Miller (Luria–Bertani) (BD Difco) at 37 °C shaking at 250 r.p.m.

**Table 1.** Bacterial strains and plasmids used in this study

Bacterial strain/Plasmid	Relevant features	Source
* Acinetobacter baumannii* ATCC17978	Type strain	ATCC
* Acinetobacter baumannii * AB258	* A. baumannii* ATCC17978::Δ*adeIJK,* Δ*adeFGH,* Δ*adeAB*	This study
* Acinetobacter baumannii * AB030	Pandrug resistant clinical isolate, MDR	[21]
* Escherichia coli* DH5α	Cloning strain	Lab collection
pMO130	Suicide plasmid, ColE1 *ori*, RK2 *ori*T, *xylE*, *sacB*, Km^R^	[13]
pFLP2	Source of Flp recombinase, Ap^R^, *sacB*,	[12]
pMO130::*ΔadeIJKGmFRT*	pMO130 with the *adeIJK* deletion fragment cloned, Gm^R^	This study
pMO130::*ΔadeFGHGmFRT*	pMO130 with the *adeFGH* deletion fragment cloned, Gm^R^	This study
pMO130::*ΔadeABGmFRT*	pMO130 with the *adeAB* deletion fragment cloned, Gm^R^	This study

**Table 2.** Oligonucleotides used in this study

Name	Amplicon size	Sequence
AdeIJK Up F	1014	GTC GAG TTC GCT TTG AAC AAT GGA AG
AdeIJK Up R	1014	TCA GAG CGC TTT TGA AGC TAA TTC GTC TGG TGC CCA AAG CTT AGC CGA CAT
AdeIJK Dn F	1056	AGG AAC TTC AAG ATC CCC AAT TCG ATC TAG TGC TGA ACT TAA AAA GCA A
AdeIJK Dn R	1056	AAA CAA CAG GGC ATA TGG AAA ATT A
AdeFGH Up F	1028	AATTTGAAGATAAACTGCTGAAATCGGCATCGGTG
AdeFGH Up R	1028	TCAGAGCGCTTTTGAAGCTAATTCGAAATGACATGAGGTGCTCCTA
AdeFGH Dn F	1068	AGGAACTTCAAGATCCCCAATTCGGGTTGGAGTAGTTAATAAAAA
AdeFGH Dn R	1068	CTAAAGAACTTTTTGGTGCAGATTACGCAAACGTT
AdeAB Up F	992	GTCGACATTCCTAAGAACTG
AdeAB Up R	992	AGGAACTTCAAGATCCCCAATTCGGTTCAATGCATCAGGGGAAA
AdeAB Dn F	1022	TCAGAGCGCTTTTGAAGCTAATTCGACCTAGTGAGTTTTTGATGT
AdeAB Dn R	1022	AGTACTACAGAAAATAGCGT
FRT Gm F	1054	CGA ATT AGC TTC AAA AGC GCT CTG A
FRT Gm R	1054	CGA ATT GGG GAT CTT GAA GTT CCT

### Generation of efflux pump deletion mutant of *A. baumannii* ATCC17978

*A. baumannii* AB258 was constructed by sequential deletion of three RND efflux pumps using a modification of previously described methods of homologous recombination [12]. Briefly, up- and downstream regions of approximately 1 kb flanking each RND pump operon were amplified. The flanking regions were joined to a gentamicin resistance cassette using sequence overlap extension. The resulting deletion cassette was cloned into the pMO130 suicide vector using blunt end cloning and a SmaI site in the vector [13]. The resulting plasmid was then introduced into *A. baumannii* ATCC17978 via electroporation [14] and homologous recombination [12] allowed for deletion of the entire RND operon. The Gm^R^ cassette was removed using a Flp recombinase encoded in pFLP2 resulting in unmarked deletion mutants [12]. This general process was performed to delete three RND operons from *A. baumannii* ATCC17978 starting with *adeIJK*, followed by *adeFGH*, and lastly, *adeAB*. The resulting strain, AB258, which was used in this study.

### Antibiotic susceptibility assays

Antibiotic susceptibility was determined using microbroth dilution method according to CLSI guidelines [15] using Mueller Hinton broth (ThermoScientific, Oxoid). Atorvastatin (Sigma Aldrich) was dissolved in DMSO with stocks stored at 10 mg ml^−1^. Tobramycin (AK Scientific) was dissolved in water at a concentration of 10 mg ml^−1^. Ciprofloxacin (BioBasic) was dissolved in water at a concentration of 10 mg ml^−1^.

### Checkerboard assays

Checkerboard assays were performed using Mueller Hinton broth (ThermoScientific, Oxoid) using a previously described method [16]. Combinations tested were Atorvastatin and Ciprofloxacin and Atorvastatin and Tobramycin. Fractional inhibitory concentrations were determined for each antibiotic according to the formula shown below:

FIC = (MIC_A in Combination_/MIC_A_) + (MIC_B in Combination_/MIC_B_)

FIC values were evaluated based on commonly set standards of FIC>4 as antagonistic; FIC>1≥4 as indifferent; FIC>0.5≤1 as additive; and FIC≤0.5 as synergistic.

## Results and discussion

A previous study had reported significant activity of atorvastatin against *A. baumannii* ATCC17978 with an MIC of 15.62 µg ml^−1^ [11]. This finding potentially has significant merit since statins are widely used pre-approved drugs and their deployment as antimicrobial agents can be rapid. However, in our study atorvastatin failed to show any activity against the pandrug resistant isolate AB030 of *A. baumannii* (Table 3). Since AB030 is known to harbour a variety of antibiotic resistance factors, and not knowing if any of these resistance factors were contributing to its lack of susceptibility to atorvastatin, we tested atorvastatin’s activity against the wild-type strain *A. baumannii* ATCC17978. However, atorvastatin failed to display any activity against *A. baumannii* ATCC17978 as well (Table 3). It should be noted that we carried out susceptibility testing using the two-fold serial dilution method, but the previous study tested the atorvastatin activity using agar medium supplemented with the drug [11]. We therefore repeated our experiments using the agar medium supplemented with up to 400 µg ml^−1^ of atorvastatin. However, we did not observe any inhibition of *A. baumannii* ATCC17978 at this concentration (data not shown).

**Table 3.** Minimum inhibitory concentrations and fractional inhibitory concentrations determined via microbroth dilution and checkerboard assays, respectively, for *A. baumannii* strains ATCC17978, AB258, and AB030

Strains	Minimum inhibitory concn (μg ml^−1^)						Fractional Inhibitory concn (FIC)	
	Individual			Combined			Atorvastatin-Tobramycin	Atorvastatin-Ciprofloxacin
	Atorvastatin	Tobramycin	Ciprofloxacin	Atorvastatin	Tobramycin	Ciprofloxacin		
ATCC17978	512	2	0.25	256	0.13	0.13	0.56	1
AB030	512	64	32	512	32	32	1.5	2
AB258	512	8	0.0156	nd			nd	nd

nd, not determined.

We then investigated the possibility if the lack of activity of atorvastatin in *A. baumannii* ATCC17978 was due to the activity of RND efflux pumps. A key contributor to the intrinsic resistance seen in *A. baumannii* is the activity of efflux pumps belonging to the resistance-nodulation-division (RND) family which are able to efflux a broad variety of substrates [17]. *A. baumannii* isolates encode for around 14 RND efflux pumps in their genome with three having been shown to be most clinically relevant: AdeIJK, AdeAB(C), and AdeFGH [17]. To determine if the activity of any of these three pumps was responsible for the lack of susceptibility of *A. baumannii* ATCC17978, we created a derivative of *A. baumannii* ATCC17978 that lacks the three above mentioned pumps. However, we did not observe any differences in the susceptibility of ATCC17978 and AB258 (efflux deletion strain) to atorvastatin, with the MIC of both strains being 512 µg ml^−1^ for atorvastatin (Table 3). These data indicate that RND efflux pumps are unlikely to play any role in the susceptibility of *A. baumannii* strains to atorvastatin.

We also explored the possibility if atorvastatin potentiates the activity of certain antibiotics against *A. baumannii *. Tobramycin and ciprofloxacin were chosen as antibiotics to test for synergy with atorvastatin based on their potential use in combination therapy with carbapenems today [5]. Unfortunately, these combinations resulted in FICs indicating indifferent effects of these combinations for the MDR *A. baumannii* AB030 and the type strain ATCC17978 with atorvastatin/ciprofloxacin. Additive effects were seen for *A. baumannii* ATCC17978 with atorvastatin/tobramycin. Our data therefore indicate that atorvastatin is not a good potentiator of the ciprofloxacin or tobramycin activity against *A. baumannii* ATCC17978 or *A. baumannii* AB030. Our findings on the lack of synergy between atorvastatin and antibiotics are similar to what has been reported previously [18] where no synergy was observed between statin with amikacin, imipenem, or minocycline [18].

To summarize, our results displaying lack of activity of atorvastatin against *A. baumannii* are somewhat surprising considering that a previous study [11] made opposite observations. The reasons behind the discrepancy of our data with previously published data is not clear to us. However, we would like to point out that genetic diversity within supposedly same strains from different laboratories has been reported previously for different bacterial species [19, 20]. Thus, it is possible that the ATCC17978 strain used in our study may contain different genetic properties from the strain previously reported. However, this will need further confirmation. Nevertheless, our data does highlight the importance of using multiple strains for testing the activity of antimicrobial compounds against bacterial species such as *A. baumannii *, that can be genetically quite diverse.

In conclusion, our findings show that the use of atorvastatin as an antimicrobial agent or as a potentiator of existing antibiotics against *A. baumannii* is likely to have limited applications.
